# Second opinions for spinal surgery: a scoping review

**DOI:** 10.1186/s12913-022-07771-3

**Published:** 2022-03-18

**Authors:** Giovanni E. Ferreira, Joshua Zadro, Chang Liu, Ian A. Harris, Chris G. Maher

**Affiliations:** 1 Institute for Musculoskeletal Health, The University of Sydney and Sydney Local Health District, Sydney, Australia; 2 School of Public Health, Faculty of Medicine and Health, The University of Sydney, PO Box M179, Missenden Road, Camperdown | NSW|, Sydney, New South Wales 2050 Australia; 3 South Western Sydney Clinical School, Liverpool Hospital, The University of New South Wales, Sydney, New South Wales Australia; 4 Ingham Institute of Applied Medical Research, Liverpool, Sydney, New South Wales Australia

**Keywords:** Low back pain, neck pain, spine surgery, Orthopaedics, Orthopaedic surgery, Health services research

## Abstract

**Background:**

Second opinions have the goal of clarifying uncertainties around diagnosis or management, particularly when healthcare decisions are complex, unpleasant, and carry considerable risks. Second opinions might be particularly useful for people recommended surgery for their back pain as surgery has at best a limited role in the management of back pain.

**Methods:**

We conducted a scoping review. Two independent researchers screened PubMed, EMBASE, Cochrane CENTRAL and CINAHL from inception to May 6th, 2021. Studies of any design published in any language were eligible provided they described a second opinion intervention for people with spinal pain (low back or neck pain with or without radicular pain) either considering surgery or to whom surgery had been recommended. We assessed the methodological quality with the Downs & Black scale. Outcomes were: i) characteristics of second opinion services for people considering or who have been recommended spinal surgery, ii) agreement between first and second opinions in terms of diagnoses, need for surgery and type of surgery, iii) whether they reduce surgery and improve patient outcomes; and iv) the costs and healthcare use associated with these services. Outcomes were presented descriptively.

**Results:**

We screened 6341 records, read 27 full-texts, and included 12 studies (all observational; 11 had poor methodological quality; one had fair). Studies described patient, doctor, and insurance-initiated second opinion services. Diagnostic agreement between first and second opinions varied from 53 to 96%. Agreement for need for surgery between first and second opinions ranged from 0 to 83%. Second opinion services may reduce surgery rates in the short-term, but it is unclear whether these reductions are sustained in the long-term or if patients only delay surgery. Second opinion services may reduce costs and healthcare use (e.g. imaging), but might increase others (e.g. injections).

**Conclusions:**

Second opinion services typically recommend less surgical treatments compared to first opinions and may reduce surgery rates in the short-term, but it is unclear whether these reductions are sustained in the long-term or if patients only delay surgery. There is a need for high-quality randomised trials to determine the value of second opinion services for reducing spinal surgery.

**Supplementary Information:**

The online version contains supplementary material available at 10.1186/s12913-022-07771-3.

## Background

Second opinions have the goal of clarifying uncertainties around diagnosis or management, particularly when healthcare decisions are complex, unpleasant, and carry considerable risks [1]. Second opinions are not uncommon and often patient-initiated: about 1 in 5 persons who visited a doctor end up seeking a second opinion [2, 3]. Second opinions can also be initiated by other parties, such as doctors and health insurers. Those initiated by doctors and health insurers may have different drivers; they may be related to reducing the provision of low-value care (i.e., care that provides little or no benefit, may cause harm, or yields marginal benefits at a disproportionately high cost) [4]. In Australia, some private health insurers currently have second opinion services, typically offered by third-parties separate to the insurer [5, 6].

Musculoskeletal conditions are amongst the most common reasons why people seek a second opinion. In an American study, requests for second opinions in orthopaedic surgery were the most common reason, representing 18% of all patient-initiated requests [7]. Similar figures were described in an Israeli study: most second opinions were sought from orthopaedic surgeons, representing 17% of all requests. In a German study, 27% of all second opinions were for spinal conditions [8]. *Reasons are not well understood but may be related to the complexity and controversial nature of spinal surgery* [9].

Second opinions might be particularly useful for people recommended surgery for their back pain as surgery has at best a limited role in the management of back pain [10, 11]. Reasons to consider a second opinion might include substantial variability in diagnoses given to people with back pain [12], indications for surgery, and risks associated with some surgical procedures that have unclear benefits (e.g. spinal fusion) [13]. Some studies have reported outcomes of second opinions for people with back pain, including a recent scoping review [14]. However this scoping review only investigated a limited number of outcomes, namely the frequency of second opinions and the discordance between first and second opinions. This review did not summarise other important outcomes such as agreement on diagnosis, agreement on surgical indication, outcomes of second opinions including surgery rates and patient-reported outcomes, the costs and healthcare use associated with second opinion programs.

Given the different design of available studies and the broad range of questions we were interested in, a scoping review was the most appropriate study design. Scoping reviews are useful for mapping the concepts underpinning a research area and the main sources and types of evidence available [15, 16]. The aims of this scoping review are to describe:

1. The characteristics of second opinion services for spinal surgery
2. Diagnostic agreement between first and second opinions
3. Agreement in treatment recommendations between first and second opinions
4. Whether they (i) reduce surgery rates, and (ii) improving patient outcomes
5. The costs and healthcare use associated with second opinion services

## Methods

### Study design and registration

We reported this scoping review per the recommendations from the PRISMA extension for Scoping Reviews (PRISMA-ScR) [17]. We followed a protocol developed a priori, however, this scoping review was not prospectively registered on PROSPERO as it does not accept scoping reviews registrations. The protocol is available in Appendix 1.

#### Searches

We searched PubMed, EMBASE and Cochrane CENTRAL from their inception to May 6th, 2021. The search terms for each database are described in Appendix 2. Two researchers (GF, JZ) independently screened studies first by reading title and abstract and then their full text using Covidence (Veritas Health Innovation, Melbourne, Australia). We conducted backward and forward citation tracking by examining the reference list of included studies and citations (using Google Scholar) to the included studies. Disagreements were resolved by discussion and consensus. If no consensus was reached a third researcher (CM) arbitrated.

### Eligibility criteria

Any study design was eligible, provided it described a second opinion intervention for people with spinal pain (low back or neck pain with or without radicular pain) either considering surgery or to whom surgery had been recommended. Second opinions could be initiated by the patient, doctor, or health insurance company. Second opinions could have been provided by an individual health professional (e.g., spine surgeon, rheumatologist), or conducted by a review board or conference. Studies describing changes in care pathways to reduce referrals to surgeons were not eligible as these do not constitute a second opinion for spinal surgery [18]. Studies published in any language were eligible.

### Data charting process and outcomes

Two independent researchers used a piloted spreadsheet to extract data from eligible studies. Data extracted included bibliographic data (year and country published), study design, characteristics of the included sample (e.g., age, sex, diagnoses), sample size, setting (e.g., tertiary outpatient specialist services), eligibility, details of the second opinion services (e.g., independence of the first and second opinions), outcomes, and results.

We extracted data for the following outcomes:

1. The characteristics of second opinion services for spinal surgery. Format could describe who initiated the service (e.g. doctor, patient, or insurer) and characteristics of the service (e.g. health professionals involved in providing the second opinion)
2. The diagnostic agreement between those providing the first and second opinions.
3. The agreement in treatment recommendations between those providing the first and second opinions. This consisted of agreement on the need for surgery and type of surgery.
4. The outcomes of second opinion services on (i) reducing rates of surgery, and (ii) improving patient-reported outcomes. These studies had to report data on the number of surgeries performed or surgery rates and have data available for at least one year following the second opinion. Data on treatment recommendations between first and second opinions were not considered for this outcome.
5. The costs and healthcare use associated with second opinion services.

### Methodological quality

We used the Downs and Black tool to appraise the methodological quality of the included studies [19]. The Downs and Black Scale has 27 items relating to quality of reporting (ten questions), external validity (three questions), internal validity (bias and confounding) (13 questions), and statistical power (one question). The scale has been shown to have high levels of agreement. Included studies were classified as “excellent” (24–28 points), “good” (19–23 points), “fair” (14–18 points) or “poor” (< 14 points) [20].

### Analyses

We summarised data using descriptive statistics when applicable. We used means and standard deviations (SD) or medians and minimum and maximum for continuous outcomes, and frequency and proportions for categorical data when appropriate.

## Results

**Fig. 1:**
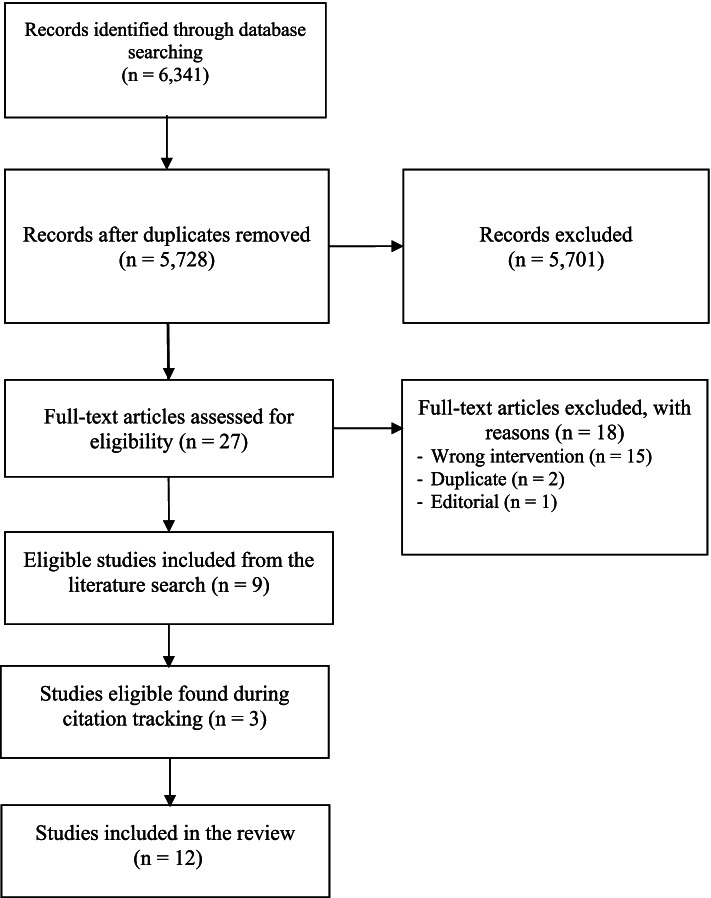
PRISMA flow diagram

We retrieved 6330 records from the electronic databases. After excluding 613 duplicates, we screened 5717 titles and abstracts. Of these, 27 studies had their full text assessed for eligibility and 9 were deemed eligible. We identified a further 3 studies upon conducting backward and forward citation tracking. We therefore included 12 studies in this review. (Fig. 1).

### Study characteristics

We identified no randomised controlled trials. All studies were observational – 8 were prospective [21–28], 3 were retrospective [29–31], and one was a cross-sectional survey study [32]. One study was a conference abstract [30]. Eight studies did not have a comparator; patients enrolled into these studies had been recommended surgery by another surgeon prior to entering the study [21, 24, 26–29, 31, 33]. Three studies had a comparator [23, 25, 30]. In two, the comparator was the period prior to the implementation of the second opinion service [23, 25]. In one, matched controls who did not seek a second opinion were used [30].

Eleven studies were classified as poor [21, 23–25, 27–33] and one was classified as fair in relation to methodological quality [26]. Across the included studies, several elements in the Downs & Black scale were poorly reported. For example, no study had a randomised design, allocation concealment, blinded study participants or personnel, adequately described the characteristics of patients lost to follow-up and conducted an a priori power analysis. Only one study collected adverse event data, described the compliance with the intervention and attempted to adjust analyses for confounding [26]. Characteristics of the included studies including their methodological quality are presented in Table 1.

**Table 1:** Characteristics of the included studies

Study, year Country Design Type	Sample size and setting	Eligibility	Intervention	Downs & Black score	Outcomes
Citation: Gamache, 2012 Country: US Design: Prospective observational study Type of second opinion: Patient-initiated	155 patients; Neurosurgery outpatient practice	No information	Consecutive patients seeking a surgical opinion for a spine problem.	4 (poor)	• Format of the service • Agreement (need for surgery)
Citation: Lenza, 2017 Country: Brazil Design: Prospective observational study Type of second opinion: Doctor-initiated	Referred for second opinion: 544 Completed stage 1: 485 Completed the full protocol: 425 Tertiary outpatient service in Brazil within a large private hospital	Inclusion All patients aged 18+ referred to the outpatient centre recommended for surgery Exclusion Spinal fractures, major scoliosis, congenital spinal deformity, spinal tumours, spondyloarthropathies, or infection”	Each patient attended two appointments with a physiatrist and an orthopaedic surgeon who did not perform spine surgery. When consensus was not reached or consensus in favour of surgery was reached, patients were seen by a spinal review board (9 senior spine surgeons and 6 neurosurgeons). The board made the final recommendation. Participants who were recommended conservative management were offered treatment at the physiotherapy outpatient service.	18 (fair)	• Format of the service • Agreement (diagnosis) • Agreement (need for surgery) • Agreement (type of surgery) • Patient-reported outcomes
Citation: Marnitz, 2019 Country: Germany Design: Retrospective observational study Type of second opinion: Patient-initiated	243 Setting unclear	No information	No information	10 (poor)	• Surgery rates
Citation: Namiranian, 2018 Country: US Design: Retrospective observational study Type of second opinion: Doctor-initiated	11 (reviewed by the spine board) 291 (pre and post spine board implementation period but not reviewed by board Veteran Affairs Maryland Health Care System	**Inclusion** Patients considered for elective lumbar spine surgery and considered at high risk of poor outcome **Exclusion** • Red flags (eg progressive lower extremity weakness, bladder disorders, fever, malignancy, intractable pain, or significant lumbar spine trauma)	A multidisciplinary spine board including orthopaedic spine surgeons, neurosurgeons, pain psychologists, physical therapists, radiologists, pain pharmacists, primary care clinicians, pain management clinicians, anaesthetists, and veteran advocacy was created. After a board discussion, a formal recommendation for the treatment plan was made.	14 (poor)	• Format of the service • Agreement (need for surgery)
Citation: Vialle, 2015 Country: Brazil Design: Prospective observational study Patient-initiated	94 Orthopaedic surgery practice	Patients aged 18–82 years who required a second opinion due to disagreement with the pre-established protocol for surgical indication	Opinions from two surgeons were compared and classified as: • Complete agreement: both surgical options were similar • Partial disagreement: minor difference in surgical indication (eg extension of procedure or number of implants). A third opinion was not needed. • Complete disagreement: there was a significant difference in surgical indication, diagnosis, need for surgery or type of procedure. Required a third opinion by another spine surgeon.	10 (poor)	• Format of the service • Agreement (need for surgery)
Citation: Yanamadala, 2017 Country: US Design: Retrospective observational study Type of second opinion: Doctor-initiated	100 Medical Centre	Inclusion Patients scheduled to undergo spine surgery involving up to three levels of fusion or unusual spinal pathology that required a multidisciplinary approach for diagnosis of treatment planning. Exclusion Not mentioned	A spine multidisciplinary conference with at least one member of the following areas: physical medicine and rehabilitation, anaesthesia pain service, neurosurgery, orthopaedic spine surgery, nursing, physical therapy, and social work. Consensus was reached on the recommendation to be given on each case. The recommendation included a decision to offer surgery or not.	11 (poor)	• Format of the service • Agreement (diagnosis) • Agreement (need for surgery)
Citation: Epstein, 2011 Country: US Design: Prospective observational study Type of second opinion: Patient-initiated	274 Neurosurgery outpatient practice	No information	Patients who had been referred for surgery by another spine surgeon and wanted a second opinion were assessed by a neurosurgeon who classified the surgical recommendations as “necessary” or “unnecessary”. There were two criteria for classifying surgeries as “unnecessary”: •No focal neurological deficits •No significant abnormal surgical pathology on imaging	3 (poor)	• Format of the service • Agreement (need for surgery)
Citation: Epstein, 2013 Country: US Design: Prospective observational study Type of second opinion: Patient-initiated	183 Neurosurgery outpatient practice	No information	Patients receiving a second opinion and for whom surgery had been recommended by another surgeon had their initial surgery recommendation classified as: • Unnecessary: surgeries recommended for pain alone, without neurological deficits, or significant radiographic abnormalities. • Wrong: Overly extensive surgeries (eg too many levels anterior, posterior, or circumferential) or performed from the wrong access route (eg anterior vs posterior vs circumferential) • Right: The neurosurgeon providing the second opinion agreed with the surgical recommendation from the previous surgeon (necessity, extent, and approach)	7 (poor)	• Format of the service • Agreement (need for surgery)
Citation: Lien, 2020 Country: US Design: Cross-sectional study Type of second opinion: patient-initiated	Online survey with 30 hospitals	N/A	N/A	8 (poor)	• Format of the service • Costs
Citation: Viola, 2013 Country: Brazil Design: Prospective observational study Type of second opinion: Doctor-initiated	419 Tertiary outpatient service in Brazil within a large private hospital	Patients recommended surgery were referred by their health insurer for a second opinion at the tertiary outpatient service	Each patient attended two appointments with a physiatrist and an orthopaedic surgeon who did not perform spine surgery. When there was no consensus or consensus that surgery was required, patients were seen by a spinal review board (9 senior spine surgeons and 6 neurosurgeons). The board made the final recommendation. Participants who were recommended conservative management were offered treatment at the physiotherapy outpatient service.	12 (poor)	• Costs
Citation: Fox, 2013 Country: US Design: Prospective observational study Type of second opinion: Insurance-initiated	No information 54 physiatrists from 33 practices providing consultations in Spine Centres of Excellence approved by the health insurer	Inclusion Any patient requiring a surgical consultation Exclusion Patients that had evidence of trauma, tumour, infection, progressive bilateral neurological findings, cauda equina syndrome, follow-up to an inpatient or emergency department evaluation by a spine surgeon	A health insurer formed a multidisciplinary advisory group to define criteria required for physiatrists to be eligible to obtain the designation of a Spine Centre of Excellence. Every patient was required to be seen by a physiatrist prior to evaluation by a spine surgeon (except if patient had any of the exclusion criteria). Surgeons were not reimbursed unless services were approved by the health insurer. After the consultation with the physiatrist, the patient could choose what care to receive (eg continue care with physiatrist, see a surgeon) without any other limitations.	10 (poor)	• Format of the service • Surgery rates • Costs • Healthcare use
**Citation: Goodman, 2016** **Country: US** **Design: Prospective observational study** **Type of second opinion:** Insurance-initiated	501 Physiatrist practices authorised to provide services to a health insurer	Inclusion People aged 18–65 with a membership with the health insurer with an episode of back pain Exclusion Serious clinical presentations or other reasons (eg surgical follow-up)	In order for a surgical consultation to be authorised by the health plan, patients were required to see a physiatrist (any) within the previous 6 months of the surgical appointment.	9 (poor)	• Format of the service • Surgery rates • Costs • Healthcare use

PROMs, patient-reported outcome measures; N/A, not applicable

### Characteristics of second opinion services

Second opinion services were either patient-initiated (*n* = 6) [21, 24, 27, 30, 32, 33], doctor-initiated (*n* = 4) [26, 28, 29, 31], or insurance-initiated (*n* = 2) [23, 25]. In four studies describing a patient-initiated second opinion service, second opinions were given by a single surgeon [21, 24, 32, 33]. In one study it was not clear who provided the second opinion [30]. Only one study described the format (e.g. in person, telehealth) by which second opinion services were delivered [32]. In a survey study with 30 neurosurgery medical centres in the US, most patient-initiated (29, 97%) second opinion services offered in-person appointments, and a small proportion (11, 37%) offered a tele-health option [32].

In the four studies describing doctor-initiated second opinion services, the final treatment recommendation typically considered the opinion of more than one health professional and did not always only involve spine surgeons in the decision (e.g., a multidisciplinary conference) [29, 31]. In two studies describing the same second opinion service, patients referred to that service were first seen by an orthopaedic surgeon (not a spine surgeon) and a physiatrist who made an independent treatment recommendation [26, 28]. When there was consensus that surgery was not required, patients were offered physiotherapy treatment. If there was consensus that surgery was required, or opinions were discordant, patients were then referred to a board of nine spine surgeons who reviewed the case and made a final recommendation. In two other studies, doctor-initiated second opinions consisted of a multidisciplinary team who made the final treatment recommendation [29, 31].

In the two studies describing insurance-initiated services, insurers adopted a mandatory consultation with a physiatrist for every patient requiring surgical evaluation with a spine surgeon [23, 25]. In one study, the service was provided by a trained physiatrist within specialised centres [23], whereas in the other study any physiatrist was eligible to provide the service [25].

### Diagnostic agreement between first and second opinions

Two studies had data on the diagnostic agreement between first and second opinions. In the study by Lenza et al. [26], diagnoses were concordant between first and second opinions for 53% of patients. Examples of diagnoses that had low concordance include cervical radiculopathy (36% agreement), lumbar radiculopathy (50% agreement), and lumbar stenosis (58% agreement) (Table 2). In contrast, Yanamadala et al. [31] had a much better agreement (96%).

**Table 2:** Data on agreement between first and second opinions

Study, year	Agreement between first and second opinions, second opinion/second opinion (%)		
	Diagnosis	Need for surgery	Type of surgery
Lenza, 2017	Overall agreement: 53% Agreement by diagnosis: - Non-specific neck pain: 4/13 (24%) - Radiculopathy (cervical): 36/99 (36%) - Radiculopathy (lumbar): 116/234 (50%) - Lumbar stenosis: 7/12 (58%) - Failed back surgery: 26/37 (70%) - Non-specific low back pain: 66/83 (80%) - Cervical myelopathy: 2/2 (100%) - Non-spinal condition: 87/0	Overall agreement: 143/425 (34%)	Agreement with the same surgical procedure recommended by the first opinion: 66/425 (16%) - Cervical fusion: 11/71 (15%) - Cervical disc arthroplasty (1 or 2 levels): 0/7 (0%) - Lumbar fusion: 25/162 (15%) - Cervical or lumbar decompression: 7/28 (25%) - Endoscopic lumbar decompression: 0/1 (0%) - Percutaneous decompression: 0/20 (0%) - Percutaneous decompression and rhizotomy: 0/1 (0%) - Stabilisation (interlaminar-interspinous): 0/8 (0%) - Nucleoplasty: 0/5 (0%) - Revision with fusion (cervical): 0/1 (0%) - Revision with fusion (lumbar): 14/22 (64%) - Steroid injection: 0/6 (0%) - Neuro-stimulator: 0/3 (0%) - Discography: 0/1 (0%) - Radiofrequency rhizotomy: 8/86 (9%) - Hardware removal: 1/1 (100%) - Not reported: 0/2 (0%)
Gamache, 2012		55%	
Namiranian, 2018		Overall agreement: 0/11 (0%)	
Yanamadala, 2017	Overall agreement: 96/100 (96%) Agreement by diagnosis - Non-spinal condition: 4/0	Overall agreement: 42/100 (42%)	
Epstein, 2011		Overall agreement: 227/274 (83%)	
Epstein, 2013		Overall agreement: 72/183 (39%)	
Vialle, 2015		Overall agreement: 58/94 (62%)	
Viola, 2013		Overall agreement: 218/399 (55%)	

### Agreement in treatment recommendations between first and second opinions

Eight studies reported data on agreement in treatment recommendations (need for surgery and type) between first and second opinions [21, 24, 26–29, 31, 33]. Agreement for need for surgery (yes/no) ranged from 0% [29] to 83% [21] (Table 2). Only one study provided data on the agreement between first and second opinions on the type of surgery [26], and showed very low agreement for commonly recommended surgeries such as lumbar fusion (15%), cervical fusion (15%), and cervical or lumbar decompression (25%).

### Surgery rates

Three studies, two insurance-initiated and one patient-initiated, had data on the outcomes of second opinion services [23, 25, 30]. In Marnitz & Wagner [30], 44% patients who received a second opinion had surgery compared to 75% of patients who did not after one year (between-group difference: 32%; 95% CI 19 to 43%).

The two studies describing an insurance-initiated service found conflicting results. Fox et al [23] found a 29% reduction in surgery rates comparing a 2-year pre and 2-year post-implementation period (2.7 vs 1.9 surgeries per 1000 plan members), whereas Goodman et al. [25], found increased surgery rates by 9% from 2008 to 2013 (68 vs 74 per 100,000 population) when implementing a similar service.

Only one study reported patient-reported outcomes from patients who had received surgery or conservative care following the second opinion recommendation [26]. One year after receiving the final recommendation from the second opinion service, those who were recommended conservative care had similar pain, disability and quality of life than those who had surgery (Table 3). These results however need to be interpreted with caution, as 72 to 78% of participants who initiated the study (*n* = 485) did not provide patient-reported outcomes. No studies compared patient-reported outcomes of people who received a second opinion versus those who did not.

**Table 3:** Data on outcomes, costs and healthcare use, effectiveness, and costs

Study, year	Surgery rates	Patient-reported outcomes	Costs	Healthcare use
Lenza, 2017		Between-group MD (95% CI) Pain (0–10): 0.5 (−0.5 to 1.6) RMDQ (0–24): 1.5 (−1.6 to 4.7) ODI (0–100): 27.4 (−7.1 to 7.3)		
Marnitz, 2019	Patients receiving surgery within 12 months, n/N (%) Second opinion: 47/108 (44%) No second opinion: 81/108 (75%) Between group difference (95% CI): 32% (19 to 43%)			
Viola, 2013			Estimated cost (USD) of treatment after second opinion versus cost initially proposed (difference): - Surgery: $1,002,826 vs $1,228,117 (difference: $225,291; −18%) - Conservative: $184,304 vs $1,840,976 (difference: $1,656,672; −90%) - Total: $1,187,294 vs 3,069,094 (difference: $1,881,800; −61%)	
Fox, 2013	Surgery rates per 1000 plan members pre/post program implementation (2006–7 vs 2008–10): 2.7 vs 1.9 (−29%)		Costs pre (2006–07) versus post-implementation (2007–08) (USD, % change): - Surgical costs per member per month: $9.75 vs $7.29 (−25%) - Total spinal-related costs per member per month: $19.7 vs $17.4 (− 12%) - Average reimbursement for surgery: $21,250 vs $22,853	Healthcare use pre (2006–07) versus post-implementation (2007–08) (% change): - Physiatrist consultations per 1000 members: 5 vs 8.5 (+ 69.5%) - New surgical consultations per 1000 members: 7.2 vs 3.7 (−48%) - Advanced imaging (CT or MRI) per 1000 members: 14 vs 11.6 (− 17.7%) - Electrodiagnostic testing, % of cases: 21% vs 24% (+ 14%) - Spinal injections, % of cases: 42% vs 44% (+ 4%)
Goodman, 2016	Surgery rates per 100,000 population pre-post program implementation (2008–2013): 68 vs 74 (+ 8%)		Costs pre (versus post-implementation (USD, % change): $4338 vs $7940 (+ 83%) Percentage of incremental costs with non-surgical care per type of service considering an average increase in cost post-implementation of $3602 per member: - Emergency: $326 (9%) - Urgent care: $1 (0%) - Observation stays: $209 (6%) - Inpatient admissions: $666 (19%) - Office visits: $379 (11%) -Physiotherapy visits: $290 (8%) - Radiology: $437 (12%) - Chiropractic: $-1 (0%) - Prescription drugs: $460 (13%) - Lumbar injections: $835 (23%)	
Lien, 2020			Mean (SD; range) cost of second opinion services across 30 hospitals in the US: $493 ($343; $90–$1300) Average cost of second opinion services provided online: $643 ($259; $100–$850)	

### Costs and healthcare use associated with second opinion services

Four studies had data on costs or healthcare use, or both (Table 3) [23, 25, 28, 32]. Lien described the costs of patient-initiated second opinion services in 30 hospitals in the US [32]. The mean (SD) cost of these services across the 30 hospitals was $US 493 per second opinion consultation (range $90–$1300). None of the services were covered by insurers. Amongst hospitals that offered online services, the mean cost was higher – $643 ($259; range $100–$850).

In Fox et al. [23], the total monthly spinal-related costs per member reduced 12% after the implementation of an insurer-initiated second opinion service. The net decrease in costs for the insurer in one year was more than $14 million. The cost reduction was driven by a decrease in surgical rates (29% reduction), surgical consultations (48% reduction), and advanced imaging such as MRI or CT scans (18% reduction). There were increased costs with physiatrist services (69% increase), electrodiagnostic testing (14% increase), and spinal injections (4% increase). The average reimbursement per surgery increased 8% (from $21,250 to $22,853). Goodman et al. only reported data on pre-surgical costs before and after the implementation of the insurance initiated second opinion service and noted an 83% increase in those costs [25]. The main drivers of the observed increase in costs were lumbar injections (23% of incremental costs), inpatient admissions (19%), prescription drugs (13%) and radiology (12%).

Viola et al. [28] reported estimated cost data. In their study, all patients had been initially referred to surgery. They estimated that there was an overall estimated reduction of 61% in costs with the second opinion service. An 18% reduction was estimated amongst those treated surgically (*n* = 54), and a 90% reduction was estimated amongst those treated conservatively (*n* = 112) (Table 3).

## Discussion

### Summary of main findings

This scoping review identified 12 observational studies describing i) characteristics of second opinion services for people considering or who have been recommended spinal surgery, ii) agreement between first and second opinions in terms of diagnoses, need for surgery and type of surgery, iii) whether they reduce surgery rates and improving patient outcomes; and iv) the costs and healthcare use associated with these services. Studies described patient-, doctor-, and insurance-initiated second opinion services. Second opinion services were typically offered by a surgeon in studies describing a patient-initiated service, by multidisciplinary teams in studies of doctor-initiated services, and by non-surgeons in insurance-initiated services.

Agreement in diagnoses and treatment recommendations (need for and type of surgery) were variable across studies. Diagnostic agreement varied from 53% [26] to 96% [31],; agreement on the need for surgery ranged from 0% [29] to 83% [21]. Second opinion services may reduce surgical rates, reduce costs and use of some healthcare resources such as advanced imaging. There might be an increase in use of other aspects of healthcare, such as expenses related to non-surgical care (e.g. injections).

### Comparison with previous studies

To the best of our knowledge ours is the first study to summarise outcomes related to second opinion services for spinal surgery. Our findings indicate that second opinions may reduce surgery rates and healthcare costs, however data are limited by the poor design and methodological quality of studies. This is in line with the literature on second opinions in other areas of healthcare. In a systematic review of patient-initiated second opinions for a range of health conditions, second opinions led to change in diagnosis, treatment, or prognosis in 10–62% of cases [1]. In a review of second opinion services for patients with cancer, substantial variability was found in the proportion of cases where changes in diagnosis, treatment recommendations or prognosis occurred: 12–69% [34]. In previous reviews, investigating second opinions for a range of medical conditions, middle-aged women and patients who were more educated were more likely to seek second opinions [35, 36]. In a review of second opinions in oncology, patients’ primary motivations for seeking a second opinion included a need for certainty, a lack of trust, dissatisfaction with communication, and/or a need for more (personalised) information [36].

A scoping review investigating the frequency and impact of second opinions has recently been published [14]. Our review provided a more comprehensive assessment of the current evidence on second opinions. Whilst that review only described frequency of patients that received a second opinion and frequency of discordant recommendations, ours described more outcomes, including agreement on diagnoses and type of surgery, outcomes of second opinions such as surgery rates, patient-reported outcomes, costs, and healthcare use associated with second opinion programs. Our findings also differentiate between different types of second opinions (e.g. doctor vs patient vs insurance-initiated). We believe this is an important distinction as the drivers for second opinions might be different depending on who initiates it. For example, whilst there is some evidence that patients seek second opinions to obtain certainty in their diagnosis or when interactions with the treating clinician have not been optimal, those initiated by doctors and health insurers may be related to reducing the provision of low-value care (i.e., care that provides little or no benefit, may cause harm, or yields marginal benefits at a disproportionately high cost).

### Meaning of the study

In our review, spinal fusion was the surgery more often considered to be unnecessary after a second opinion [21, 26, 33]. Second opinion services may be a promising intervention to curb the rise in rates [37–39] of spinal fusion, a costly surgery [10] with questionable benefits compared to structured non-surgical care for people with spinal pain due to degenerative conditions [13]. These services may reduce the use of spinal fusion by mainly two mechanisms: improving the uptake of conservative care and reducing the complexity of surgical procedures. Zadro et al. [40] found that 1 in 6 patients did not receive any course of physiotherapy, and 1 in 6 patients had between 1 and 8 sessions prior to undergoing lumbar fusion. Försth et al. [41] found that lumbar fusion added to decompression in patients with lumbar stenosis (with or without degenerative spondylolisthesis) had negligible effects on pain or disability, but increased hospital length of stay by an average of 3 days, and more than doubled costs ($5400 vs $12,200).

Whether second opinion services may reduce surgery rates remains uncertain. In Fox et al. [23], which reported a reduction in surgery rates, physiatrists who saw patients prior to a surgery consultation were part of specialised centres to treat spinal problems, whereas any physiatrist could provide the service in Goodman et al., which reported an 9% increase in surgical rates [25]. This indicates that specific training of clinicians providing these services may be an important component to ensure services are successful. The length of follow-up in both studies also offer potentially interesting insights on why findings from both studies differ. While Fox et al. had data from 2006 to 07 and 2008–10 [23], Goodman had 6 years of data and did report a 9.2% decrease in surgery rates in 2011. However, by 2013 surgery rates had already gone up again and were 9% higher than when the study began in 2008. One hypothesis suggested by Goodman et al. is that the service only delayed patients getting surgery - hence the transitory reductions in surgery rates. This transitory change was also associated with increased costs with healthcare services that may also have questionable value for back pain (e.g. spinal injections, imaging) [11]. The study also showed a 13% increase in the use of prescription drugs, some of which are known to be ineffective for back pain and increase the risk of adverse events, such as opioids and antidepressants [22, 42]. Whether delaying surgery is a good outcome for patients and health systems remains an unanswered question which should be answered by future studies.

### Strengths and limitations

Strengths of our study include a comprehensive literature search across various electronic databases, study screening and data extraction processes that were conducted in duplicate by independent reviewers in accordance with best practices, and assessment of methodological quality (also conducted in duplicate by two independent reviewers).

Most studies included in this review (*n* = 11) had poor methodological quality, and we could not find any randomised controlled trials investigating the effectiveness of second opinion services for reducing surgery rates and improving patient-reported outcomes. This limitation is particularly relevant for the outcomes we studied: surgery rates, patient-reported outcomes, costs and healthcare use. The lack of a randomised trial assessing the effectiveness of second opinion services is a major gap in the literature that needs to be addressed.

Very few studies provided actual data on surgical rates after a second opinion, which could be considered a limitation of the literature. Most studies had a prospective observational design and focused on describing the agreement between first and second opinions. A disagreement between the two opinions, and a second opinion not recommending surgery might not be enough for patients to avoid surgery as patients might have sought additional opinions and had surgery with a different surgeon. For example, 58% of patients who received a final recommendation of conservative care were lost to follow-up and no information about whether they had surgery or not was available in the study by Lenza et al. [26] Better documentation of decisions made by first and second opinion services and adequate follow-up strategies of patients to ascertain whether or not they had surgery are important aspects that need to be addressed by future studies.

## Conclusion

Different formats of second opinion services for reducing spinal surgery have been reported. Second opinion services typically recommend less surgical treatments compared to first opinions, particularly for spinal fusion. Second opinion services may reduce surgery rates in the short-term, but it is unclear whether these reductions are sustained in the long-term or if patients only delay surgery. There are no studies comparing health outcomes between those who received versus did not receive a second opinion. There is a need for high-quality randomised trials to determine the value of second opinion services for reducing spinal surgery.

## Supplementary Information

**Additional file 1.**

**Additional file 2.**

## Data Availability

All data generated or analysed during this study are included in this published article.
