# Supplementary material for: Second opinions for spinal surgery: a scoping review
Source: BMC Health Serv Res. 2022 Mar 18;22:358. doi: 10.1186/s12913-022-07771-3 (PMC8932184; doi:10.1186/s12913-022-07771-3)
Supplement: Supplementary file 1 — Additional file 1. [file 12913_2022_7771_MOESM1_ESM.docx]

**Second opinions for spinal surgery: a scoping review**

Giovanni E Ferreira^1^, Joshua Zadro^1^, Chang Liu^1^, Ian A Harris^1,2,3^, Chris G Maher^1^

1. Institute for Musculoskeletal Health, The University of Sydney and Sydney Local Health District, Sydney, Australia

2. South Western Sydney Clinical School, Liverpool Hospital, The University of New South Wales, Sydney, New South Wales, Australia

3. Ingham Institute of Applied Medical Research, Liverpool, Sydney, New South Wales, Australia

**Appendix 1. Scoping review protocol [v3, May 2021]**

**Background**

Second opinions have the goal of clarifying uncertainties around diagnosis or management, particularly when healthcare decisions are complex, unpleasant, and carry considerable risks.1 Second opinions are not uncommon and often patient-initiated: about one in 5 persons who visited a doctor end up seeking a second opinion.2,3 Second opinions can also be initiated by other parties, such as doctors and health insurers. Those initiated by doctors and health insurers may have different drivers; they may be related to reducing the provision of low-value care (i.e., care that provides little or no benefit, may cause harm, or yields marginal benefits at a disproportionately high cost).4 In Australia, some private health insurers currently have second opinion programs, typically offered by third-parties.5,6

Musculoskeletal conditions are often the most common reasons why people seek a second opinion. In an American study, requests for second opinions in orthopaedic surgery were the most common reason, representing 18% of all patient-initiated requests.7 Similar figures were described in an Israeli study: however most second opinions were sought from orthopaedic surgeons, representing 17% of all requests. In Germany, spinal conditions represented 27% of patients who sought a second opinion.8

Second opinions might be particularly useful for people recommended surgery for their back pain; as surgery has at best a limited role in the management of back pain. Reasons to consider a second opinion might include the substantial variability in the diagnoses given to people with back9, the indications for surgery, and risks associated with some surgical procedures that have unclear benefits (e.g. spinal fusion).10 Some studies have reported outcomes of second opinion programs for people with back pain, but there has not been any attempts to summarise the evidence available for a range of outcomes of second opinion programs designed for people with back pain to whom surgery has been recommended.

We are aware of some studies investigating the agreement between first and second opinions and the consequences of such programs in terms of reducing recommendations for spinal surgery and reducing rates of surgery. However, we are also interested in mapping other concepts that underpin this research area, such as the characteristics of second opinion programs described in the published studies and whether there are data available on the costs of these programs from different perspectives (e.g. society, health systems), patient satisfaction with the programs and studies reporting patient-reported outcomes, among others. Given our interest in mapping a potentially broad area of research, a scoping review design was chosen.11

We will report this scoping review per the recommendations from the PRISMA extension for Scoping Reviews (PRISMA-ScR).12

Pending the availability of studies, the aims of this scoping review are to describe:

1. The characteristics of second opinion services for spinal surgery

2. The reasons why people with back pain (and doctors and insurers) seek a second opinion when considering surgery or when recommended surgery

3. The agreement between diagnosis and treatment recommendations obtained between first and second opinions

4. The effectiveness of second opinion programs for spinal surgery for (i) reducing recommendations for spinal surgery, (ii) reducing rates of surgery, and (iii) improving patient-reported outcomes

5. The costs associated with second opinion programs

6. Patient satisfaction following a second opinion for spinal surgery

**METHODS**

***Study design***

Scoping review

***Searches***

We will search Pubmed, EMBASE and Cochrane Central from their inception until May 6th, 2021. We plan to update searches once more prior to submission. The search terms for each database is described in Appendix 1. Two, from a panel of three researchers (GF, CL, JZ) will independently screen studies first by reading title and abstract and then their full-text. We will also conduct backward and forward citation tracking by examining the reference list of included studies and citations of those studies. Disagreements will be resolved by discussion and consensus. If no consensus is reached a third researcher (CM) will arbitrate.

***Eligibility criteria***

Any study design will be eligible, as long as it covers:

• Patients with back pain (low back or neck pain) with or without radicular symptoms

• Describes a second opinion intervention for people either considering surgery or to whom surgery has been recommended. Second opinions can be patient, doctor or health insurance-initiated. Second opinions might be done by one individual health professional (e.g., a spine surgeon or another medial specialty such as rheumatology), conducted by a review board or conference.

Studies describing changing care pathways to reduce referrals to surgeons (eg where patients are redirected from a consultation with a spine surgeon to another health professional) are not eligible as these do not constitute a second opinion for spinal surgery.13

***Data charting process and outcomes***

Two independent researchers will use a dedicated spreadsheet to extract data from eligible studies. Data extracted will include bibliographic data (year and country published), study design, characteristics of the included sample (e.g., age, sex, diagnoses) second opinion, sample size, setting (e.g., tertiary outpatient specialist services), eligibility, details of the second opinion program (e.g., independence of the first and second opinions), outcomes and results.

We have chosen, a priori, seven outcomes of interest:

1. The characteristics of second opinion services for spinal surgery

2. The reasons why people with back pain (or their doctors or insurers) seek a second opinion when considering surgery or when recommended surgery

3. The diagnostic agreement obtained by first and second opinions

4. The agreement between first and second opinions regarding treatment recommendations

5. The effectiveness of second opinion programs for spinal surgery for (i) reducing recommendations for spinal surgery, (ii) reducing rates of surgery, and (iii) improving patient-reported outcomes

6. The costs associated with second opinion programs

7. Patient satisfaction following a second opinion for spinal surgery

However, given our goal of mapping all the available evidence around second opinion programs for spinal surgery, we might add other outcomes.

***Analyses***

We will summarise data using descriptive statistics when applicable. Data will be presented descriptively for each outcome – using means and standard deviations or median and interquartile range for continuous outcomes, and frequency and proportions for categorical data.

**REFERENCES**

1. Payne VL, Singh H, Meyer AN, Levy L, Harrison D, Graber ML. Patient-initiated second opinions: systematic review of characteristics and impact on diagnosis, treatment, and satisfaction. *Mayo Clin Proc.* 2014;89(5):687-696.

2. Wagner TH, Wagner LS. Who gets second opinions? *Health Aff (Millwood).* 1999;18(5):137-145.

3. Shmueli L, Shmueli E, Pliskin JS, et al. Second Medical Opinion: Utilization Rates and Characteristics of Seekers in a General Population. *Med Care.* 2016;54(10):921-928.

4. Könsgen N, Prediger B, Bora A-M, et al. Analysis of second opinion programs provided by German statutory and private health insurance – a survey of statutory and private health insurers. *BMC Health Services Research.* 2021;21(1):209.

5. HCF. Second opinion service. https://www.hcf.com.au/members/access-medical-resources/second-opinion-services. Published 2021. Accessed.

6. BUPA. Best Doctors. https://www.bupa.com.au/health-insurance/best-doctors. Published 2021. Accessed.

7. Meyer AN, Singh H, Graber ML. Evaluation of outcomes from a national patient-initiated second-opinion program. *Am J Med.* 2015;128(10):1138 e1125-1133.

8. Weyerstraß J, Prediger B, Neugebauer E, Pieper D. Results of a patient-oriented second opinion program in Germany shows a high discrepancy between initial therapy recommendation and second opinion. *BMC Health Services Research.* 2020;20(1):237.

9. Herzog R, Elgort DR, Flanders AE, Moley PJ. Variability in diagnostic error rates of 10 MRI centers performing lumbar spine MRI examinations on the same patient within a 3-week period. *Spine J.* 2017;17(4):554-561.

10. Chou R, Baisden J, Carragee EJ, Resnick DK, Shaffer WO, Loeser JD. Surgery for low back pain: a review of the evidence for an American Pain Society Clinical Practice Guideline. *Spine (Phila Pa 1976).* 2009;34(10):1094-1109.

11. Tricco AC, Zarin W, Ghassemi M, et al. Same family, different species: methodological conduct and quality varies according to purpose for five types of knowledge synthesis. *J Clin Epidemiol.* 2018;96:133-142.

12. Tricco AC, Lillie E, Zarin W, et al. PRISMA Extension for Scoping Reviews (PRISMA-ScR): Checklist and Explanation. *Ann Intern Med.* 2018;169(7):467-473.

13. Fourney DR, Dettori JR, Hall H, Härtl R, McGirt MJ, Daubs MD. A systematic review of clinical pathways for lower back pain and introduction of the Saskatchewan Spine Pathway. *Spine (Phila Pa 1976).* 2011;36(21 Suppl):S164-171.
