# Supplementary material for: Second opinions for spinal surgery: a scoping review
Source: BMC Health Serv Res. 2022 Mar 18;22:358. doi: 10.1186/s12913-022-07771-3 (PMC8932184; doi:10.1186/s12913-022-07771-3)
Supplement: Supplementary file 2 — Additional file 2. [file 12913_2022_7771_MOESM2_ESM.docx]

**Appendix 2. Search strategy**

| **Pubmed (1264 records)**  (low back pain OR back pain OR sciatica OR Back Injuries[MeSH Terms]) OR (backache[Title/Abstract] OR back problems[Title/Abstract]) AND "second opinion"[Title/Abstract] OR opinion[Title/Abstract] OR mandatory[Title/Abstract] OR authorization[Title/Abstract] OR board[Title/Abstract] OR panel[Title/Abstract] OR "multidisciplinary board"[Title/Abstract] OR "multidisciplinary panel"[Title/Abstract] OR "review panel"[Title/Abstract] OR "multidisciplinary conference"[Title/Abstract] OR conference[Title/Abstract] OR multidisciplinary[Title/Abstract] AND  (surgery OR Orthopedic Procedures OR Diskectomy OR Laminectomy OR Arthrodesis OR Spinal Fusion OR Decompression, Surgical[MeSH Terms]) OR (orthopaedic surgery[Title/Abstract] OR elective surgery[Title/Abstract] OR operation[Title/Abstract] spine surgery[Title/Abstract] OR spinal surgery[Title/Abstract] OR lumbar surgery[Title/Abstract]) |
| --- |
| **EMBASE (n = 4152)**  1 exp low back pain/ or exp backache/ or back pain.mp. or lumbago.mp.  2 exp patient referral/ or second opinion.mp.  3 mandatory.mp.  4 exp prior authorization/ or authorization.mp.  5 board.mp.  6 panel.mp.  7 multidisciplinary board.mp.  8 multidisciplinary panel.mp.  9 review panel.mp.  10 conference.mp.  11 surgery  12 exp spine surgery/ or exp surgery/  13 exp spine fusion/  14 exp discectomy/  15 exp laminectomy/  16 operation.mp.  17 2 or 3 or 4 or 5 or 6 or 7 or 8 or 9 or 10  18 11 or 12 or 13 or 14 or 15 or 16  19 1 and 17 and 18 |
| **CENTRAL (n = 510 [507 without 3 editorials])**  (low back pain OR back pain OR sciatica OR back injury OR back problem OR backache):ti,ab,kw AND (second opinion OR opinion OR board OR panel OR multidisciplinary OR conference):ti,ab,kw AND (surgery OR orthopaedic procedures OR diskectomy OR laminectomy OR arthrodesis OR spinal fusion OR decompression surgery OR orthopaedic surgery OR elective surgery OR operation OR spine surgery OR spinal surgery OR lumbar surgery):ti,ab,kw" |
| **CINAHL (n = 11)**  S1 (MH "Back Pain+") OR (MM "Low Back Pain")  S2 (MH "Sciatica")  S3 (MH "Back Injuries+")  S4 "second opinion"  S5 (MH "Multidisciplinary Care Conference (Iowa NIC)")  S6 "review panel"  S7 (MH "Orthopedic Surgery+")  S8 (MH "Diskectomy")  S9 (MH "Laminectomy")  S10 (MH "Arthrodesis+")  S11 (MH "Spinal Fusion")  S12 (MH "Decompression, Surgical+")  S13 S1 OR S2 OR S3  S14 S4 OR S5 OR S6  S15 S7 OR S8 OR S9 OR S10 OR S11 OR S12  S16 S13 AND S14 AND S15 |
